# T2 mapping of the heart with high temporal and spatial resolution using a radial double inversion fast spin-echo pulse sequence with view sharing

**DOI:** 10.1186/1532-429X-14-S1-O112

**Published:** 2012-02-01

**Authors:** Maria Altbach, Tomoe Barr, Jaspreet Singh, Bujji Ainapurapu, Dipak Kc, Scott Squire, Jean-Philippe Galons, Chuan Huang, Aiden Abidov

**Affiliations:** 1 Radiology, University of Arizona, Tucson, AZ, USA; 2 Biomedical Engineering, University of Arizona, Tucson, AZ. USA; 3 Medicine, University of Arizona, Tucson, AZ, USA

## Summary

A double inversion radial fast spin echo (DIR-RADFSE) has been developed to obtain T2 maps of the heart with high-temporal and spatial resolution from data acquired in a single breath hold. The method allows for the quantitative assessment of inflammation in the heart.

## Background

While DE imaging is considered a gold standard in the evaluation of myocardial scar/viability in patients with old MI or cardiomyopathy, a few recent publications demonstrate a higher diagnostic sensitivity of T2-weighted techniques in patients with Non-STEMI and myocarditis where changes in T2-weighting are due to inflammation in the myocardium leading to edema (Abdel-Aty H, JACC 53:1194, 2009; Tilak GS, Invest Radiol 43:7, 2008).

Thus, recently there has been great interest in measuring T2, the parameter responsible for contrast in T2-weighted images (Giri S, JCMR 11:56, 2009; Kim D Magn Reson Med 62:300, 2009). Most proposed methods, however, do not have adequate spatial and temporal resolution for detecting subtle changes in the myocardium T2 or are affected by artifacts caused by motion and flow.

Our group developed a Double Inversion Radial Fast Spin Echo (DIR-RADFSE) sequence that yields TE images and T2 maps of the heart, with fewer motion and flow artifacts compared to conventional methods. DIR-RADFSE yields data for 16 perfectly registered TE images (in plane resolution ~ 1.3-1.6 mm^2^). The TE images are used for the voxel-wise generation T2 maps. The high temporal resolution afforded by DIR-RADFSE allows for accurate T2 estimation. The high spatial resolution enables detecting T2 changes in small areas.

## Methods

**Figure 1:**
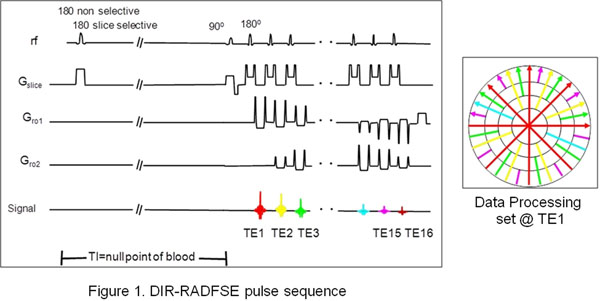


DIR-RADFSE (Fig. 1) was implemented on a 1.5T GE Signa MRI scanner. Data were acquired in one breath-hold using ETL=16, 256 views, 256 readout points, BW=±32 kHz, TR=1RR, NEX=1.

A high-resolution anatomical image is reconstructed from the full radial k-space data set. High-resolution images at various TE_eff_ are generated from the same k-space data by view sharing using data at a specific TE in the center of k-space. Data at TE≠TE_eff_ are incorporated in a progressive manner from the center to the outer part of k-space (Altbach MI Magn Reson Med 54:549, 2005). T2 maps are generated from the TE_eff_ images.

## Results

**Figure 2:**
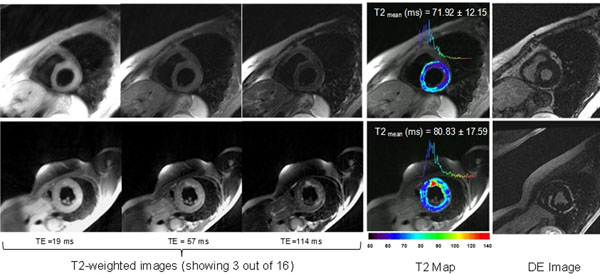


Figure 2 shows TE images (3 out of the 16 TE images shown) and the corresponding colorized T2 map of the LV overlaid onto the anatomical image. Histograms showing the T2 distribution in the LV are also included with the mean T2 values and standard deviation. The delayed enhancement (DE) images are also shown.

The top row images show a T2 map for a normal patient. The bottom row images show an area of nonspecific inflammation in the RV insertion point (T2> 120 ms) in a patient with hypertrophic cardiomyopathy and dense ventricular ectopy. Of interest, DE images in the same patient were unremarkable.

## Conclusions

A method for fast T2 mapping of the heart has been presented. The method yields data with high temporal and spatial resolution thus allowing the detection of T2 changes within the heart indicative of inflammation.

## Funding

NIH grant R01HL085385 and the Edward and Virginia Madden Award.

